# A call for parental monitoring to improve condom use among secondary school students in Dar es Salaam, Tanzania

**DOI:** 10.1186/1471-2458-12-1061

**Published:** 2012-12-09

**Authors:** Linda B Mlunde, Krishna C Poudel, Bruno F Sunguya, Jessie K K Mbwambo, Junko Yasuoka, Keiko Otsuka, Omary Ubuguyu, Masamine Jimba

**Affiliations:** 1 Department of Community and Global Health, Graduate School of Medicine, The University of Tokyo, 7-3-1, Hongo, Bunkyo-ku, Tokyo, 113-0033, Japan; 2 Department of Public Health, School of Public Health and Health Sciences, University of Massachusetts, Amherst, 316 Arnold House, 715 North Pleasant St, Amherst, MA, USA; 3 Department of Psychiatry and Mental Health, Muhimbili University of Health and Allied Sciences, P. O. BOX 65001, Dar es Salaam, Tanzania

**Keywords:** Young people, Sexual behaviors, Condom use, Parental monitoring, Tanzania

## Abstract

**Background:**

The number of people newly infected with human immunodeficiency virus (HIV) has been decreasing in sub-Saharan Africa, but prevalence of the infection remains unacceptably high among young people. Despite the alarming pervasiveness of the virus, young people in this region continue to engage in risky sexual behaviors including unprotected sexual intercourse. In developed countries, parents can play important roles in protecting young people from such behaviors, but evidence regarding the impact of parental involvement is still limited in sub-Saharan Africa. Therefore, we conducted this study to examine the magnitude of risky sexual behaviors and the association of parental monitoring and parental communication with condom use at last sexual intercourse among secondary school students in Dar es Salaam, Tanzania.

**Methods:**

We conducted this cross-sectional study among 2,217 male and female students aged 15 to 24 years from 12 secondary schools in Dar es Salaam. From October to November 2011, we collected data using a self-administered questionnaire. Multiple logistic regression analyses were conducted to examine the association of parental monitoring and parental communication with condom use at last sexual intercourse, adjusting for potential confounders.

**Results:**

A total of 665 (30.3%) secondary school students reported being sexually active within the year prior to data collection. Among them, 41.7% had multiple sexual partners, 10.5% had concurrent sexual partners, and 41.1% did not use a condom at last sexual intercourse. A higher level of parental monitoring was associated with increased likelihood of condom use at last sexual intercourse among male students (AOR: 1.56, 95% CI: 1.05-2.32; *p* = 0.03) but not among female students (AOR: 1.54, 95% CI: 0.71-3.37; *p* = 0.28). The association between parental communication and condom use at last sexual intercourse among both male and female students was not statistically significant.

**Conclusions:**

A high level of parental monitoring is associated with more consistent condom use among male students in Dar es Salaam, Tanzania -- many of whom have engaged in high-risk sexual behaviors such as multiple sexual partnerships, concurrent sexual partnerships, and unprotected sexual intercourse in the past one year. Interventions should thus be strengthened to reduce multiple sexual partnerships, concurrent sexual partnerships, and to improve parental monitoring among such students toward increasing condom use.

## Background

Despite a notable decrease in new HIV infections in sub-Saharan Africa from 2.2 million in 2001 to 1.9 million in 2010 [1], HIV prevalence among young people remains unacceptably high. In 2009, the number of young people aged 15–24 years living with HIV in sub-Saharan Africa was 3.8 million [2]. This figure represents 76% of the global burden of the disease among young people [2].

Studies have reported risky sexual behaviors as a common practice among young people in sub-Saharan Africa. Young people in this region frequently engage in pre-marital sexual intercourse, with consequences such as unplanned pregnancy [3], sexually transmitted infections (STIs) [4], and HIV/acquired immunodeficiency syndrome (AIDS) [5,6]. Against the prevailing cultural norms in Sub-Saharan Africa, such young people also tend to engage in having multiple sexual partnerships [4-6], concurrent sexual partnerships [7] and unprotected sexual intercourse [3-6].

Tanzania, one of the resource-limited countries in sub-Saharan Africa, has been experiencing a high burden of HIV/AIDS [8]. The major route of HIV transmission in the country is heterosexual intercourse [9]. Although HIV prevalence has decreased in the general population, it remains as high as 4.3% among people aged 20–24 years [9]. Even under such conditions of high prevalence, young people often engage in risky sexual behaviors such as early sexual debut, multiple sexual partnerships and unprotected sexual intercourse [6,10,11]. For example, only about 50% of women and 46% of men aged 15–19 years and 47.6% of women and 60.4% of men aged 20–24 years reported using a condom at last sexual intercourse in 2009 [12].

Parental monitoring may reduce risky sexual behaviors among young people in Tanzania, as in other countries [13,14]. Parental monitoring involves knowing children’s whereabouts and with whom they are associating when they are not at home or at school [14,15]. In this way, parents may set rules to guide their children into safer behaviors. In contrast, poor parental monitoring may increase the influence of deviant peers in young people’s lives [16]. In Tanzania, young people spend most of their time every day outside the home, unsupervised by their parents, either at school or on public transport to and from home. During such times, they may associate particularly with their peers and other non parental figures. This might constitute a vital time for young people to engage in risky activities such as substance use and sexual activities. Moreover, some adolescents have a tendency to sneak out at night and go to discos, where they may engage in sexual activities [17].

Communication between parents and their children on sexual and reproductive health (SRH) matters promotes safer sexual practices among young people [18]. Parents can be a source of information and may guide young people on the proper norms and values related to SRH [18]. However, the idea of adults talking about SRH with young people has remained a sensitive issue -- regarded as something immoral, even -- in Tanzania [19]. Parents thus find it difficult to talk to their children about SRH matters due to cultural norms. A qualitative study found that only a few parents talk about sexual matters with their children [20]. Such communication is often abstracted and focuses on adverse outcomes of sexual behaviors such as HIV/AIDS and unwanted pregnancy rather than practical life skills [20]. For example, when parents talk about HIV/AIDS, they expect their children to understand all matters related to SRH [20]. According to the same study, only a few parents talk to their children about condom use.

In such an environment, we sought to assess whether increased parental monitoring and parental communication may bring about protective effects regarding sexual behaviors, particularly in terms of condom use, among young people in Tanzania. The roles of parental monitoring and communication in protecting young people from risky sexual behaviors have been previously studied in the developed country context [14,15]. Despite a growing body of literature in SRH in sub-Saharan Africa [13,17], we could not find any study that has examined the association of parental monitoring with condom use in the region. Moreover the association of parental monitoring and parental communication with condom use has not been examined among young people in Tanzania. Therefore, the objectives of this study were to examine the magnitude of the problem of risky sexual behaviors and the association of parental monitoring and parental communication with condom use at last sexual intercourse among secondary school students in Dar es Salaam, Tanzania.

## Methods

### Study design, area and participants

We conducted this cross-sectional study among secondary school students in the Dar es Salaam region from October to November 2011. In 2006, the HIV prevalence in this region was 9.0% in the general population and 3.1% among people aged 15–24 years [9]. In addition, 34.0% of people aged 15–24 years in the region reported having sexual intercourse in the same year [9]. Among the participants, 68.2% of females and 76.2% of males reported using a condom at last sexual intercourse [9].

Tanzania’s education system includes seven years of primary education and six years of secondary education. For secondary school education, the six years are further divided into two levels: four years for ordinary level (Form One through Form Four) and two years for advanced level (Form Five and Form Six). Some students in Tanzania may also start school at a later age; as a result, the age of students in secondary schools ranges from 14 years to over 24 years.

The Dar es Salaam region has a total of 125 public and 180 private secondary schools. Of them, we selected six public and six private secondary schools. All of the selected schools were located within close proximity of the city centre. From these schools, we sampled students of Form One through Form Six, excluding Form Four students. Students of Form Four were on vacation during the fieldwork period of this study because they had just completed their final national examinations. We recruited all the students of other Forms (n = 2,251) who were present on the day of the data collection, were aged 15–24 years, and agreed to participate in the study.

We calculated the sample size using Epi Info version 6. We set the ratio of the number of students unexposed to those exposed to parental communication as 2.3:1.0 [21]. For the expected percentage of condom use among students unexposed to parental communication, we used a rate of 43% [10]. The expected percentage of condom use among students exposed to parental communication, meanwhile, was estimated at 51%. We used a power of 80% and a 95% confidence interval (CI) to yield a minimum required sample size of 1,498 students. To counteract the effect of missing data, we decided to recruit all of the 2,268 students who were present in the classroom during our fieldwork. However, we excluded seventeen students from the study; fifteen of these excluded students were aged more than 24 years and two students were not permitted by their parents to participate in this study.

### Measurements

#### Sexual behaviors

To measure sexual behaviors, we asked the students if they had ever had sexual intercourse. In case of a positive response, we further asked them the age at which they had sexual intercourse for the first time, if they had ever used a condom or used it at last sexual intercourse, and if they had ever had sexual intercourse with a sex worker. A participant who reported experience of sexual intercourse was considered to be sexually active. We also asked them the number of sexual partners [22,23] and the time they first and last had sexual intercourse with their partners to assess concurrent sexual partnerships using the Joint United Nations Programme on HIV/AIDS (UNAIDS) questionnaire [24]. A participant was considered to have had a concurrent sexual partnership if s/he reported more than one ongoing sexual partnership at a point in time within the past one year.

#### Parental monitoring

We assessed parental monitoring with a six-item Silverberg’s Parental Monitoring Scale [25]. Using this scale, we asked the students how often they informed their parents about their whereabouts and with whom they are spending time when they are not at home or at school. Items were scored from 1 (*never*) to 5 (*always*). In this study, a parent was referred to as either a biological parent or any other adult living with a student, as his or her guardian. A score lower than the median value was considered as low parental monitoring. Cronbach’s alpha for the Silverberg’s Parental Monitoring Scale in our study was 0.82.

#### Parental communication

We measured parental communication with a five-item Parent-Adolescent Communication Scale [26]. Using this scale, we asked the students how often they talked with their parents on several SRH matters. Such aspects include sexual intercourse; condom use; and prevention against sexually transmitted infections, HIV/AIDS and pregnancy. Items were scored from 1 (*never*) to 4 (*often*). A score lower than the median value was considered as low parental communication. Cronbach’s alpha for the Parent-Adolescent Communication Scale in our study was 0.86.

#### Self-esteem

To assess self-esteem, students filled out a ten-item Rosenberg Self-Esteem Scale [27]. This is a Likert scale previously used in Tanzania, with responses ranging from 0 (*strongly disagree*) to 3 (*strongly agree*). Item numbers 2, 5, 6, 8, and 9 were reverse-scored to rank from 0 (*strongly agree*) to 3 (*strongly disagree*) [28]. A score lower than the median value was considered as low self-esteem. Cronbach’s alpha in our study for the Rosenberg Self-Esteem Scale was 0.64.

#### Delinquency

We assessed delinquency with a Problem Oriented Screening Instrument for Teenagers (POSIT) scale [29]. The scale has 10 subscales with a total of 139 items. From this scale, we used a sixteen-item aggressive behavior/delinquency subscale previously used in South Africa [30]. A score lower than the median value was considered as low delinquency. Cronbach’s alpha in our study for the POSIT scale was 0.72.

#### Perceived stress

To assess perceived stress, students filled out a fourteen-item Perceived Stress Scale [31]. This Likert scale, previously used in Zimbabwe, has items scored from 0 (*never*) to 4 (*very often*). Item numbers 4, 5, 6, 7, 9, 10 and 13 were reverse-scored to rank from 0 (*very often*) to 4 (*never*) [32]. A score lower than the median value was considered as low perceived stress. Cronbach’s alpha in our study for the Perceived Stress Scale was 0.70.

#### Substance use

We assessed substance use with modified questions from a previous study [33]. For example, one of the questions included was as follows: “During the past 12 months, did you use any kind of illicit drugs?” Possible answers were either *yes* or *no* for the use of any of a number of listed illicit drugs such as marijuana, heroin, and others.

#### School connectedness

To assess school connectedness, students filled out a six-item School Connectedness Scale [34]. This scale has been previously used in Nigeria [35]. A score lower than the median value was considered as low school connectedness. Cronbach’s alpha in our study for the School Connectedness Scale was 0.76.

#### Economic status

We used a proxy wealth index to measure economic position based on Tanzania Demographic and Health Survey (TDHS) questionnaire contents including source of drinking water, toilet type, properties owned, type of fuel used for cooking, source of energy for lighting, and food security [23]. We dichotomized the items to make binary variables, using Principal Component Analysis (PCA) to reduce such variables from 40 to 13, loaded as factor 1. We used the factor loadings as variable weights totaled to yield household’s weighted wealth index. We divided the total weighted wealth index into terciles to give low, middle, and high levels of economic status.

#### Socio-demographic characteristics

We also measured age, sex, level of education, family structure, and parents’ or guardians’ employment status.

### Data collection

We hired two research assistants for the fieldwork. These research assistants received a one-day training orientation covering questionnaire contents and data collection procedures. We pre-tested the questionnaire among 30 students from a school not included in the current study. Based on the pre-test, we then modified a few of the questions. We collected data using a structured questionnaire in the Swahili language. Students filled out the questionnaire in halls or classes in the absence of their teachers. Before distributing the questionnaires, we explained to the students how to respond to the questions. We supervised the filling out of questionnaires and collected them at the end of their administration.

### Data analysis

Of the surveyed 2,251 students, we excluded the questionnaires of 34 students due to missing information. As a result, we analyzed the data of 2,217 students using both descriptive and logistic regression analyses, stratified by sex.

For descriptive analyses, we used chi-square and independent sample T-tests to describe the socio-demographic characteristics and sexual behaviors of the students. We conducted bivariate logistic regression analysis to examine the relationship between condom use at last sexual intercourse and other variables. Then, we conducted multiple logistic regression analysis to examine the association of parental monitoring and parental communication with condom use at last sexual intercourse, after adjusting for confounders. The confounding variables included in the model were those associated with condom use at a *p ≤* 0.2 in bivariate analysis. The variables included in the model did not have multicollinearity; the variance inflation factor values of all the variables were less than 2. We conducted all analyses using PASW 18 (SPSS Inc., Chicago, Illinois, USA).

### Ethical consideration

This study was approved by the Research Ethics Committee of The University of Tokyo, Japan and the Senate Research and Publications Committee of the Muhimbili University of Health and Allied Sciences, Tanzania. Participation was voluntary and confidentiality was ensured. As in previous comparable studies in Tanzania [21] and England [36], we sent letters to the parents explaining the details of the study. Parents were free to have their child opt out of the study. Students who participated in this study also provided verbal informed consent.

## Results

### General characteristics

Table 1 shows the characteristics of all students who participated in this study. Out of the 2,217 participating students, 1,335 (60.2%) were male students. A majority of students were aged less than 20 years (63.3%). Compared to female students, a higher proportion of male students were in the advanced level of secondary education (80.8% vs. 63.8%), were living with a guardian(s) other than the biological parent(s) (30.0% vs. 26.7%), had ever had sexual intercourse (53.1% vs. 20.6%), and reported sexual intercourse within the past one year (39.3% vs. 16.8%). A higher proportion of female students than male students were from households of high economic status according to the wealth index.

**Table 1:** General characteristics of the students

	**Total (n = 2,217)**	**Male (n = 1,335)**	**Female (n = 882)**	***p*-value**
	**n (%)**	**n (%)**	**n (%)**	
**Age (yrs)**				
< 20 years	1404 (63.3)	715 (53.6)	689 (78.1)	< 0.001
≥ 20 years	813 (36.7)	620 (46.4)	193 (21.9)	
**Secondary school class**				
Ordinary-level	575 (25.9)	256 (19.2)	319 (36.2)	< 0.001
Advanced-level	1642 (74.1)	1079 (80.8)	563 (63.8)	
**Religion**				
Christian	1447 (65.3)	875 (65.5)	572 (64.9)	0.77
Other	770 (34.7)	460 (34.5)	310 (35.1)	
**Family structure**				
Both biological parents	1213 (54.8)	734 (55.0)	479 (54.4)	0.03
One biological parent	367 (16.6)	200 (15.0)	167 (18.9)	
Other	635 (28.6)	400 (30.0)	235 (26.7)	
**Father's or guardian's job**				
Employed	1992 (95.5)	1197 (94.8)	795 (96.5)	0.10
Unemployed	94 (4.5)	65 (5.2)	29 (3.5)	
**Wealth Index**				
Low	713 (32.2)	466 (34.9)	247 (28.0)	< 0.001
Middle	760 (34.3)	459 (34.4)	301 (34.1)	
High	744 (33.6)	410 (30.7)	334 (37.9)	
**Ever had sexual intercourse**				
Yes	891 (40.2)	709 (53.1)	182 (20.6)	< 0.001
No	1326 (59.8)	626 (46.9)	700 (79.4)	
**Sexually active in the past one year**				
Yes	665 (30.3)	518 (39.3)	147 (16.8)	< 0.001
No	1529 (69.7)	800 (60.7)	729 (83.2)	

Table 2 shows the characteristics of the 665 students who were sexually active in the past one year. A higher proportion of male students aged 20 years or above (53.1%) were sexually active compared to female students aged 20 years or above (42.9%). Compared to female students, a higher proportion of male students in advanced-level schooling were sexually active (86.3% vs. 76.9%), and had used drugs (13.9% vs. 6.8%) and/or cigarettes (12.1% vs. 5.4%) in the past one year. A total of 411(61.8%) sexually active students had low levels of parental monitoring. No statistically significant difference was observed between male and female students on parental monitoring. About 46% of sexually active students had low levels of parental communication. A higher proportion of male students had low levels of parental communication than did female students (48.1% vs. 37.4%).

**Table 2:** Descriptive characteristics of sexually active students in the past one year

	**Total (n = 665)**	**Male (n = 518)**	**Female (n = 147)**	***p*-value**
	**n (%)**	**n (%)**	**n (%)**	
**Age (yrs)**				
< 20 years	327 (49.2)	243 (46.9)	84 (57.1)	0.04
≥ 20 years	338 (50.8)	275 (53.1)	63 (42.9)	
**Secondary school class**				
Ordinary-level	105 (15.8)	71 (13.7)	34 (23.1)	0.01
Advanced-level	560 (84.2)	447 (86.3)	113 (76.9)	
**Religion**				
Christian	461 (69.3)	359 (69.3)	102 (69.4)	1.00
Other	204 (30.7)	159 (30.7)	45 (30.6)	
**Family structure**				
Both biological parents	317 (47.7)	256 (49.4)	61 (41.8)	0.20
One biological parent	105 (15.8)	82 (15.8)	23 (15.8)	
Other	242 (36.5)	180 (34.8)	62 (42.4)	
**Father's or guardian's job**				
Employed	596 (94.8)	466 (95.1)	130 (93.5)	0.60
Unemployed	33 (5.2)	24 (4.9)	9 (6.5)	
**Wealth Index**				
Low	207 (31.1)	165 (31.9)	42 (28.6)	0.31
Middle	231 (34.7)	184 (35.5)	47 (32.0)	
High	227 (34.2)	169 (32.6)	58 (39.4)	
**Alcohol use in the past one year**				
No	439 (66.0)	338 (65.3)	101 (68.7)	0.50
Yes	226 (34.0)	180 (34.7)	46 (31.3)	
**Drug use in the past one year**				
No	583 (87.7)	446 (86.1)	137 (93.2)	0.03
Yes	82 (12.3)	72 (13.9)	10 (6.8)	
**Cigarette use in the past one year**				
No	584 (87.8)	445 (85.9)	139 (94.6)	0.01
Yes	81 (12.2)	73(12.1)	8 (5.4)	
**Self-esteem**^**a**^				
Low	310 (46.8)	246 (47.6)	64 (43.8)	0.48
High	353 (53.2)	271 (52.4)	82 (56.2)	
**Parental monitoring**^**a**^				
Low	411 (61.8)	327 (63.1)	84 (57.1)	0.22
High	254 (38.2)	191 (36.9)	63 (42.9)	
**Parental communication**^**a**^				
Low	304 (45.7)	249 (48.1)	55 (37.4)	0.03
High	361 (54.3)	269 (51.9)	92 (62.6)	
**Delinquency**^**a**^				
Low	166 (25.0)	133 (25.7)	33 (22.8)	0.54
High	497 (75.0)	385 (74.3)	112 (77.2)	
**Perceived stress**^**a**^				
Low	251 (37.7)	196 (37.8)	55 (37.4)	1.00
High	414 (62.3)	322 (62.2)	92 (62.6)	
**School connectedness**^**a**^				
Low	324 (48.7)	255 (49.2)	69 (46.9)	0.69
High	341 (51.3)	263 (50.8)	78 (53.1)	

^**a**^ Self-esteem, parental monitoring, parental communication, delinquency, perceived stress, and school connectedness were coded as *low* if the score was lower than median value.

### Risky sexual behaviors

Table 3 shows the risky sexual behaviors reported by sexually active students in the past one year. Male students had a lower mean age of sexual debut than did female students (16.7 years vs. 17.3 years). A higher proportion of male students had ever had sexual intercourse with a commercial sex worker compared to female students (12.7% vs. 1.4%). About 41% of students did not use a condom at last sexual intercourse; condom use was not significantly different between male and female students. Compared to female students, a higher proportion of male students reported multiple sexual partners in their lifetime (76.2% vs. 51.4%) and within the past one year (46.1% vs. 25.9%). Compared to female students, a higher proportion of male students reported concurrent sexual partnerships in their lifetime (14.5% vs. 5.4%) and in the past one year (12.2% vs. 4.8%).

**Table 3:** Risky sexual behaviors of students who were sexually active in the past one year

	**Total (n = 665)**	**Male (n = 518)**	**Female (n = 147)**	
	**n (%)**	**n (%)**	**n (%)**	***p*-value**
**Age at sexual intercourse debut [mean yrs] (S.D)**		[16.7] (2.5)	[17.3] (2.1)	0.003
**Ever had sexual intercourse with a sex worker**				
Yes	68 (10.2)	66 (12.7)	2 (1.4)	< 0.001
No	596 (89.8)	452 (87.3)	144 (98.6)	
**Ever use of condom**				
No	114 (17.3)	93 (18.1)	21 (14.5)	0.37
Yes	544 (82.7)	420 (81.9)	124 (85.5)	
**Condom use at last sexual intercourse**				
No	271 (41.1)	213 (41.4)	58 (40.0)	0.83
Yes	388 (58.9)	301 (58.6)	87 (60.0)	
**Number of lifetime sexual partners**				
1 partner	194 (29.3)	123 (23.8)	71 (48.6)	< 0.001
≥ 2 partners	468 (70.7)	393 (76.2)	75 (51.4)	
**Number of sexual partners in the last one year**				
1 partner	388 (58.3)	279 (53.9)	109 (74.1)	< 0.001
≥ 2 partners	277 (41.7)	239 (46.1)	38 (25.9)	
**Lifetime concurrency**				
No	582 (87.5)	443 (85.5)	139 (94.6)	0.01
Yes	83 (12.5)	75 (14.5)	8 (5.4)	
**Concurrency in the past one year**				
No	595 (89.5)	455 (87.8)	140 (95.2)	0.02
Yes	70 (10.5)	63 (12.2)	7 (4.8)	

### Factors associated with condom use

Factors associated with condom use at last sexual intercourse in bivariate logistic regression analysis are shown in Table 4. In the multiple logistic regression analysis, a high level of parental monitoring was associated with condom use at last sexual intercourse among male students (adjusted odds ratio [AOR]: 1.56, 95% CI: 1.05-2.32; *p* = 0.03). The association of parental communication with condom use at last sexual intercourse among male students was not statistically significant (AOR: 1.22, 95% CI: 0.85-1.76; *p* = 0.29). Male students were less likely to have used a condom at last sexual intercourse if they belonged to a religion other than Christianity (AOR: 0.65, 95% CI: 0.43-0.98; p = 0.04), lived with a guardian(s) other than a biological parent(s) (AOR: 0.64, 95% CI: 0.43-0.98; *p* = 0.04), had a higher level of delinquency (AOR: 0.63, 95% CI: 0.40-0.99; *p* = 0.04), and had used alcohol in the past one year (AOR: 0.64, 95% CI: 0.41-0.98; *p* = 0.04). Among female students, the association of condom use at last sexual intercourse with parental monitoring (AOR: 1.54, 95% CI: 0.71-3.37; *p* = 0.28) and parental communication (AOR: 1.60, 95% CI: 0.76-3.37; *p* = 0.22) was not statistically significant.

**Table 4:** Associations of condom use at last sexual intercourse among students who were sexually active in the past one year

	**Male**				**Female**			
	**OR (95% CI)**	***p*-value**	**AOR**^**b**^**(95% CI)**	***p*-value**	**OR (95% CI)**	***p-*value**	**AOR**^**c**^**(95% CI)**	***p*-value**
**Age (yrs)**								
<20	1.00		1.00		1.00		1.00	
≥20	1.18(0.83-1.68)	0.36	1.15(0.79-1.68)	0.46	0.81(0.42-1.58)	0.54	0.91(0.40-2.06)	0.82
**Secondary school class**								
Ordinary-level	1.00				1.00		1.00	
Advanced-level	0.91(0.54-1.52)	0.71	-		0.40(0.17-0.96)	0.04	0.39(0.14-1.13)	0.08
**Religion**								
Christian	1.00		1.00		1.00			
Other	0.74(0.51-1.08)	0.12	0.65(0.43-0.98)	0.04	1.15(0.56-2.36)	0.71	-	
**Wealth Index**								
Low	1.00				1.00			
Middle	1.06(0.69-1.63)	0.78	-		1.34(0.57-3.15)	0.51	-	
High	0.97(0.63-1.50)	0.88			1.19(0.53-2.68)	0.67		
**Family structure**								
Both parents	1.00		1.00		1.00			
One parent	0.93(0.56-1.56)	0.79	1.03(0.60-1.75)	0.92	1.48(0.54-4.02)	0.45	-	
Other	0.64(0.43-0.94)	0.02	0.64(0.43-0.98)	0.04	1.25(0.60-2.58)	0.55		
**Self-esteem**^**a**^								
Low	1.00				1.00			
High	1.09(0.76-1.55)	0.64	-		1.21(0.62-2.37)	0.58	-	-
**Parental monitoring**^**a**^								
Low	1.00		1.00		1.00		1.00	
High	1.91(1.31-2.79)	0.001	1.56(1.05-2.32)	0.03	1.77(0.89-3.52)	0.10	1.54(0.71-3.37)	0.28
**Delinquency**^**a**^								
Low	1.00		1.00		1.00			
High	0.53(0.35-0.81)	0.004	0.63(0.40-0.99)	0.04	0.80(0.36-1.78)	0.58	-	-
**Perceived stress**^**a**^								
Low	1.00		1.00		1.00		1.00	
High	0.70(0.49-1.02)	0.06	0.84(0.57-1.23)	0.37	0.47(0.23-0.96)	0.04	0.74(0.32-1.72)	0.49
**Parental communication**^**a**^								
Low	1.00		1.00		1.00		1.00	
High	1.30(0.92-1.85)	0.14	1.22(0.85-1.76)	0.29	1.94(0.97-3.85)	0.06	1.60(0.76-3.37)	0.22
**School connectedness**^**a**^								
Low	1.00		1.00		1.00		1.00	
High	1.26(0.89-1.79)	0.20	1.06(0.72-1.54)	0.78	1.63(0.83-3.18)	0.15	1.54(0.73-3.22)	0.26
**Drug use in the past one year**								
No	1.00		1.00		1.00			
Yes	0.63(0.38-1.03)	0.07	0.98(0.52-1.85)	0.96	1.36(0.33-5.66)	0.67	-	
**Alcohol use in the past one year**								
No	1.00		1.00		1.00		1.00	
Yes	0.60(0.42-0.87)	0.01	0.64(0.41-0.98)	0.04	0.52(0.25-1.05)	0.07	0.61(0.27-1.38)	0.24
**Cigarette use in the past one year**								
No	1.00		1.00		1.00			
Yes	0.65(0.39-1.06)	0.09	0.92(0.49-1.74)	0.79	1.71(0.32-9.11)	0.53	-	
**Sexual partners in the past one year**								
1 partner	1.00				1.00		1.00	
≥ 2 partners	0.84(0.59-1.20)	0.34	-		0.54(0.25-1.14)	0.11	0.91(0.38-2.20)	0.84
**Concurrency in the past one year**								
No	1.00				1.00			
Yes	0.73(0.43-1.24)	0.24	-		0.88(0.19-4.10)	0.87	-	

^**a**^ Self-esteem, parental monitoring, delinquency, perceived stress, parental communication and school connectedness were coded as *low* if the score was lower than median value.

^**b**^ Adjusted for age, religion, family structure, parental monitoring, delinquency, perceived stress, parental communication, drug use, alcohol use, and cigarette use in the past one year.

^**c**^ Adjusted for age, class, parental monitoring, perceived stress, parental communication, school connectedness, alcohol use, and sexual partners in the past one year.

## Discussion

This study found that a high proportion of secondary school students engage in risky sexual behaviors in Dar es Salaam, Tanzania. About 40% of students who reported being sexually active in the past one year had multiple sexual partnerships. Our study also found that 10.5% of secondary school students had experienced concurrent sexual partnerships. Despite such high proportions of multiple and concurrent sexual partnerships, 41.1% of young people who had sexual intercourse in the past one year did not use a condom at last sexual intercourse. A higher level of parental monitoring among male students in this study was significantly associated with the condom use at last sexual intercourse.

A high proportion of young people in this study engaged in multiple sexual partnerships. The proportion of secondary school students engaging in such risky behavior reported in our study was higher than that recorded among young people of the general population in Tanzania [12]. For example, 13.9% of men and 3.3% of women aged 15–24 years were reported to have multiple sexual partnerships in a population survey conducted in 2010. Having multiple sexual partnerships increases the risk of being infected with STIs and HIV [37]. This study also found a high proportion of concurrent sexual partnerships among secondary school students in Dar es Salaam. Though the importance of concurrency as a main driver of the HIV epidemic in Africa may still be debatable [38], it nevertheless remains a high-risk behavior that needs to be addressed. Concurrency is said to fuel HIV transmission more than serially monogamous relationships [39]. This is because, when one person in the network is infected with HIV, this person places the whole sexual network at a risk of such infection.

Although condom use reduces the risk of transmission of HIV and other STIs [40], a high proportion of students in our study did not use such protection at last sexual intercourse. Clearly, this is an important concern, as the prevalence of HIV/AIDS in the general population is high in Dar es Salaam, Tanzania. The goal to reach zero new HIV infections [41] may be far from reach with such trends of involvement in risky sexual behaviors. However, our results regarding the association between parental monitoring and condom use present an important area for intervention that may be useful to reduce risky sexual behaviors, particularly among male students.

A high level of parental monitoring among male students of this study was associated with condom use at last sexual intercourse. Young male students who are closely monitored may feel especially loved and cared for by their parents. For that reason, they may refrain from risky sexual behaviors to avoid disappointing their parents, particularly when it may result in STIs and pregnancy. Other male students may refrain from risky sexual behaviors to avoid being punished by their parents when undesired outcomes such as pregnancy occur [17]. In addition, parental monitoring may reduce engagement in risky behaviors directly or indirectly by improving social skills and reducing the influence of deviant peers [42]. In the USA, one study also found an association between parental monitoring and condom use among adolescents [14], though another study among young adults in the USA did not find such an association [43]. In Tanzania, young people typically live with their parents until they get married. Therefore, parents in Tanzania may exert influence in their lives for a longer duration than in other countries such as the USA.

In this study, the association of parental monitoring with condom use at last sexual intercourse among female students was not statistically significant. This may be due to issues of gender inequality. In Tanzania, female students receive a higher level of monitoring from parents and from community members compared to male students [17]. Also, despite high levels of parental monitoring, its effect among female students may not be significant when they lack autonomy in sexual relationships. For example, previous studies have reported the limited influence women have on sexual relationships, which may compromise their powers to negotiate condom use [44,45].

The lack of association between parental communication and condom use at last sexual intercourse among both male and female students may be due to the context of Tanzanian and African culture. In this context, talking about SRH matters is considered taboo [21]. Furthermore, some parents who talk to their children on such matters may lack adequate knowledge and proper communication skills [21]. Therefore, even where such communication does occur, it may be of poor quality and lack significant effects to promote safer sexual behaviors such as condom use among young people.

Male students who live with caregivers other than their own biological parents were less likely to use condoms. Such caregivers may not care greatly nor have firm control on sexual behavior of male students in their care. Our results on this theme are consistent with a previous study done in Cameroon [46].

Male students who used alcohol and had high level of delinquency were less likely to use a condom at last sexual intercourse. Young people who consume alcohol may lose their control to a certain extend and thus be more likely to engage into other risky behaviors such as unprotected sexual intercourse. A previous review study undertaken in a developing country context reported a similar association between condom use and alcohol consumption [47].

Our results should be interpreted in the light of two key limitations. First, self-reporting of sexual behaviors might have introduced the risk of a social desirability bias. To minimize such risk, we ensured privacy and made the data collection as comfortable as possible through the absence of their teachers in the classes or halls in which the questionnaires were administered. Second, our results may not be generalized to all young people in the country particularly in the rural areas. However, these findings may be useful to populations of the urban areas and areas with people of similar background in the Tanzanian context and within other countries in the region.

## Conclusion

A high proportion of secondary school students who reported being sexually active in the past one year had multiple sexual partnerships and concurrent sexual partnerships, while condom use at last sexual intercourse remained low in Dar es Salaam, Tanzania. Among male students, a high level of parental monitoring was associated with condom use at last sexual intercourse. Our findings highlight the importance of interventions to reduce risky sexual behaviors among secondary school students in Tanzania. Such interventions should aim to reduce multiple sexual partnerships and concurrent sexual partnerships, and to increase condom use. Interventions to improve parental monitoring of secondary school students are of paramount importance to increase condom use, particularly among male students. Through such interventions, we need to encourage parents to be cognizant of where their children are after school and when they go out at night. In addition, they should know where their children go and with whom their children are going to be associating before their children go out. Finally, parents should talk with their children about the plans their children have with their friends.

## Abbreviations

HIV: Human Immunodeficiency Virus; STIs: Sexually Transmitted Infections; HIV/AIDS: Human Immunodeficiency Virus/Acquired Immunodeficiency Syndrome; SRH: Sexual and Reproductive Health; UNAIDS: Joint United Nations Programme on HIV/AIDS; POSIT: Problem Oriented Screening Instrument for Teenagers; TDHS: Tanzania Demographic and Health Survey; PCA: Principal Component Analysis; PASW: Predictive Analytics Software; SPSS: Statistical Package for the Social Sciences; AOR: Adjusted Odds Ratio; CI: Confidence Interval; USA: United States of America.

## Competing interests

The authors declare that they have no competing interests.

## Authors' contributions

LBM conceived the study, contributed to the study design, conducted the fieldwork, analyzed the data, and wrote the article. KCP helped conceptualize the study and contributed to the study design, statistical analysis, interpretation of the data and revisions of the article. BFS contributed to the study design, statistical analysis, interpretation of the data and revisions of the article. JKKM contributed to the study design, preparation of fieldwork logistics, supervision of data collection and revisions of the article. JY contributed to the study design and revisions of the article. KO contributed to study design and revisions of the article. OU contributed to oversight of fieldwork logistics and revisions of the article. MJ monitored the study progress and contributed to the study design and revisions of the article. All authors read and approved the final manuscript.

## Pre-publication history

The pre-publication history for this paper can be accessed here:

http://www.biomedcentral.com/1471-2458/12/1061/prepub
